# Concurrent Effects of Exercise and Curcumin on Spatial Learning and Memory in Sensitized Male Mice Following Morphine Administration

**DOI:** 10.31661/gmj.v8i0.1072

**Published:** 2019-12-29

**Authors:** Laleh Elhampour, Mohammad Ali Azarbayjani, Mohammad Nasehi, Maghsoud Peeri

**Affiliations:** ^1^Department of Exercise Physiology, Tehran Central Branch, Islamic Azad University, Tehran, Iran; ^2^Cognitive and Neuroscience Research Center (CNRC), Amir-Almomenin Hospital, Tehran Medical Sciences Branch, Islamic Azad University, Tehran, Iran

**Keywords:** Curcumin, Dimethyl Sulfoxide, Exercise, Memory, Central Nervous System Sensitization

## Abstract

**Background:**

Exercise and Curcumin have positive effects on spatial memory and cognition independently. The present study aims to investigate whether the combination of ineffectual dosage of these factors can affect cognition and as a solvent if DMSO is involved in Curcumin effects.

**Materials and Methods:**

Male NMRI mice (1-month-old) swam (1 week) for 60 minutes (5days/week) and injected with morphine (2.5 mg/ml/kg, intraperitoneal) for five days. Spatial learning and memory were assessed by Moris Water Maze test on the 10th day after stopping morphine injection.

**Results:**

The findings revealed that exercise, dimethyl sulfoxide (DMSO), and Curcumin increased memory formation induced by 2.5 mg/ml/kg morphine. DMSO+exercise decreased memory formation induced by morphine, but curcumin +exercise could return the effect of DMSO on the cognition.

**Conclusion:**

As a solvent, DMSO had independent effects on memory, which lead to memory impairment in combination with exercise. Therefore, considering its unpredictable effects on cognitive performance, it should be replaced with another solvent or might be used carefully in behavioral experiments.

## Introduction

Addiction causes medical and social problems. On the other hand, drugs change the brain and neural system [1, 2]. Meanwhile, chronic opiate addiction leads to mental and physical disorders [3]. Also, it affects reward pathways in the brain, decreases neural proliferation, and neurogenesis, especially at hippocampus, which causes memory and cognitive impairments [2, 4]. Depression is a mental intolerance seen in many addicted persons, and it can affect dopamine (DA) levels extremely, which leads to retention and recognition deficit, at the same time [5, 6]. Exercise promotes neurogenesis, increases brain-derived neurotrophic factor (BDNF) and TrKB, improves synaptic plasticity and memory, decreases morphine dependency, alters long term potentiation, and some of the protein concentrations [7]. Forced or voluntary exercise decreases negative effects of morphine on behavior and emotion; furthermore, it can decrease depression and anxiety [8]. It is shown that active compounds derived from Turmeric have antioxidant, anti-inflammatory, and therapeutic effects [9, 10]. Curcumin is the effective constitutive of Turmeric that divided into three main forms: bisdemethoxycurcumin, dimethoxycurcumin, and Curcumin [9]. Curcumin (1, 7-bis [4-hydroxy 3-methoxy phenyl]-1,6-heptadiene- 3,5-dione,) is a polyphenol with neuroprotective effects [9-11]. Curcumin reduces oxidative damage in the brain, prevents the decline of malondialdehyde and superoxide dismutase activities, regulates BDNF [12, 13] and extracellular signal-regulated kinase [13, 14]. Moreover, it decreases neurotoxicity effects in the hippocampus [10-12, 14], stimulates neurotic cells proliferation and neurogenesis [14], and adjusts acetylcholinesterase (AChE) activity in the brain [9, 10, 12]. Curcumin prevents IƘBα (nuclear factor of kappa light polypeptide gene enhancer in B-cells inhibitor, alpha) phosphorylation and therefore inhibits NF-ƘB (nuclear factor kappa-light-chain-enhancer of activated B cells) [14]. Meanwhile, Curcumin increases postsynaptic density protein 95/ synaptic associated protein 90 (PSD-95/ SAP 90) expression and availability [10, 14]. Therefore it facilitates ACh releasing and conduction of signals that lead to memory enhancement and learning and cognitive improvement. DMSO is a solvent for Curcumin, while, some studies mentioned that it is neuroprotective and improves memory and central nervous system (CNS) performance. DMSO can inhibit neuron death induced by glutamic exotoxins. Additionally, it can affect pyruvate metabolism that eventually leads to ATP increasing [15-17]. However, in some literature, it is shown that DMSO leads to amnesia and cognitive impairment [18]. However, some researches showed DMSO has no significant effect on spatial memory [19-21]. So, there is a paradigm about the role of DMSO on memory and learning performance. This research was designed to investigate the concurrent effect of exercise and Curcumin on spatial learning, memory in sensitized male mice and whether DMSO as a solvent involves in Curcumin responses.

## Materials and Methods

### 1. Animals

First of all, at the beginning of the experiment, male NMRI mice weighed 19-21 gr. Then, they were housed in plexiglass cages (every 4 mice were housed in a cage) with food and water available at 24 ± 20C, 50 % humidity on 12:12 h light/ dark cycle. Adaptation to the environment was occurred seven days before the experiment began. Mice did not access to food and water 1 hour before the experiment. All animal procedures were done in accordance with the National Institutes of Health Guide for the Care and Use of Laboratory Animals (NIH publications No. 80–23).

### 2. Drugs

Morphine sulfate (Merck, Germany) soluble proportion with 0.9 % NaCl (normal saline) was injected 2.5 mg/kg for five days. Curcumin (Merck, Germany) at a dose of 100 mg/kg was injected, and it was dissolved in 2.5 ml pure DMSO (Merck, Germany). All injections were administered intraperitoneally. Simultaneously, control mice were syringed saline in a volume of 10 ml/kg body weight.

### 3. Morris Water Maze (MWM) Apparatus

Spatial learning and memory were assessed in a tank (52 cm diameter, 147 cm height) that it was filled with water (25 ± 20 C) to a depth of 38 cm. There was a transparent circular platform (15 cm diameter, 37 cm height) that it was hidden 0.5 cm beneath the water surface. Furthermore, the platform was placed in a fixed shaft in one of the quadrants, and it remained at the same location during trials. There was a fixed camera above the center of the tank to record videos from trials and probs that was connected to a computer.

### 4. Experimental Groups and Study Design

There were seven groups including eight mice per group. The study consisted of two experimental stages. The first stage was the adaptation period, and the second stage was designed to specify the effect of exercise and Curcumin on behavior formation induced by morphine.

4.1. Adaptation

This stage was designed to create compatibility with exercise or environmental stress, which it took 14 days. Swimming (exercise groups) or sitting down on a platform in the water (sedentary groups) started at 5 minutes and increased to 60 minutes after two weeks.

4.2. Swimming

During one week, exercise groups swam 60 minutes within five days per week. We eliminated mice who were stayed under the surface of the water for more than 10 seconds. They were injected by saline for five days; also, their spatial learning and memory were assessed by MWM 10 days after stopping morphine injection.

4.3. Swimming + Morphine

Mice swam for one week, and they were injected by five days morphine with exercise; meanwhile, their spatial memory was evaluated ten days after stopping morphine injection.

4.4. DMSO+ Morphine

Mice were injected by DMSO (injection was in the same volume as Curcumin). During one week, subjects were put on a platform on the surface of the water (60 minutes), five days per week (same protocol as swimming group) and they were injected by morphine for five days with the sedentary situation. Then spatial learning and memory of subjects were evaluated by MWM 10 days (sensitization period) after stopping morphine injection.

4.5. Curcumin+ Morphine

Mice were injected by Curcumin. DMSO was used as the solvent. The sedentary position on a platform on the surface of the water was the same protocol as the swimming group for the subject, and they were injected by morphine for five days with the sedentary situation. Then spatial learning and memory of subjects were evaluated by MWM 10 days after stopping morphine injection.

4.6. DMSO+ Swimming+ Morphine

Subjects were injected by DMSO (as volume as Curcumin), and they swam for one week. Then, as they were swimming, they were received morphine and DMSO within five days. MWM was performed on 10th days after stopping morphine injection.

4.7. Curcumin+ Swimming+ Morphine

Curcumin was injected, and they swam for one week. DMSO was used as a solvent. Then, as they were swimming, they were received morphine and Curcumin within five days. MWM was performed on 10th day (sensitization period) after stopping morphine injection.

### 5.Measurement of Spatial Learning and Memory

MWM is a method to assess spatial learning and memory in rodents, and its protocol took place within two days. In the first day, the subject must find a hidden platform under water and hold it to form spatial memory for 20 seconds. This operation repeated for every quadrant two times, so it included two blocks and eight trials. There were 30 and 60 seconds rest between every trial and block, respectively. In the second day, the platform was removed; besides, distance and time of movement in the platform quadrant were assessed. In the first day, the process to find a hidden platform to escape latency from swimming path length were defined as spatial learning; additionally, in the second day, time and distance spent in the target quadrant were determined as spatial memory. After the probe test, the platform was covered by a shiny sheet, and the observation time was recorded as visibility time [1, 22, 23]. Data of spatial learning and memory were recorded by Video Tracking software (Borj Sanat Azma Co, Tehran. Iran).

### 6. Statistical Analysis

Differences between groups were analyzed with independent T-test, repeated measure, and One-way ANOVA (Tukey’s test post hoc). Analyses were done by SPSS version 16 software (IBM Company, New York, U.S.A).

## Results

### Learning Assessment

**Figure 1:**
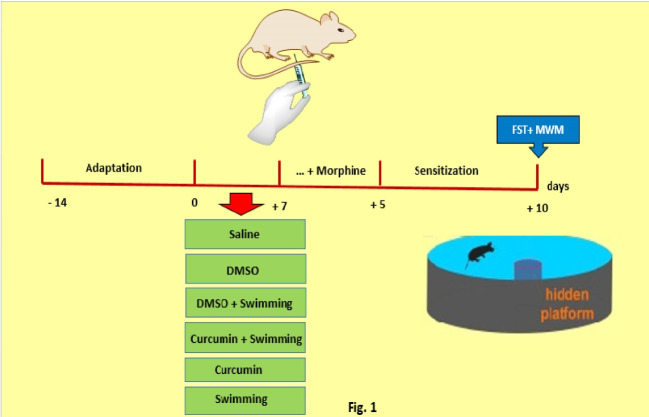
Experimental design

**Figure 2:**
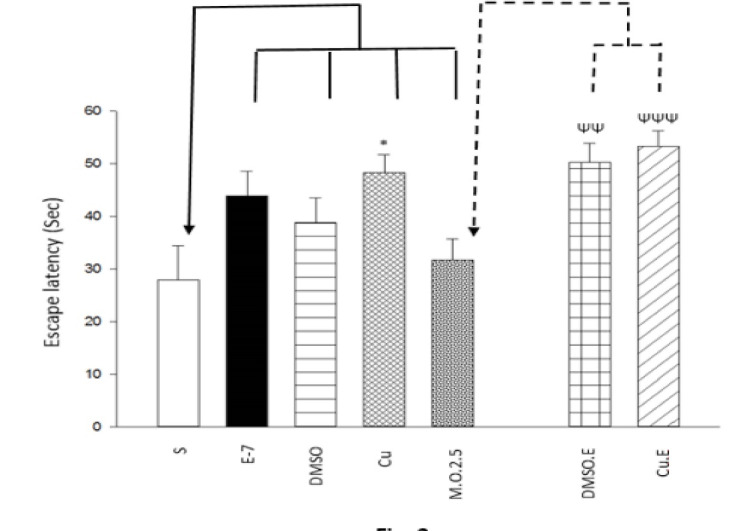
The effect of concurrent usage of exercise and Curcumin on escape latency to find the hidden platform. Subjects were divided to seven groups; saline, exercise (one week), DMSO, Curcumin, morphine (2.5 mg/ml/kg), DMSO+exercise, Curcumin+exercise. * P<0.05 as comparison between saline and exercise, DMSO, Curcumin, morphine. ΨΨP<0.01 and ΨΨΨP<0.001 as comparison between morphine and DMSO+exercise, Curcumin+exercise.

To assess the learning acquisition, One way ANOVA and Tukey post hoc test indicated that swim training, DMSO injection, and dependency to 2.5 mg/ml/kg morphine did not change escape latency (F [6,49]=4.750, P>0.05; Figure-1) and path length (F [6,49]=3.341, P>0.05; Figure-2) to find the hidden platform, but Curcumin injection decreased escape latency (F [6,49]=4.750, P<0.05; Figure-1); meanwhile, there isn’t the same result for path length (F [6,49]=3.341, P>0.05; Figure-2). Furthermore, in terms of dependency to 2.5 mg/ml/kg morphine, Independent T-test revealed that DMSO or Curcumin injection with 1 week swimming increased escape latency (t= - 3.392, P<0.05; Figure-1) and (t= - 4.344, P<0.05; Figure-1), respectively and decreased path length (t=2.927, P<0.05; Figure-2) and (t=2.480, P<0.05; Figure-2), respectively. It could conclude that DMSO and Curcumin injection with 1-week exercise can alter the responses (escape latency and path length to find the hidden platform) induced by morphine (2.5 mg/ml/kg) on spatial learning in the sensitization term.

### Memory Assessment

**Figure 3:**
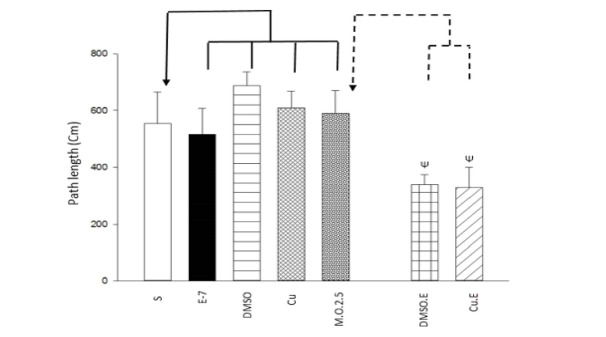
The effect of concurrent usage of exercise and Curcumin on path length to find the hidden platform. Subjects were divided to seven groups; saline, exercise (one week), DMSO, Curcumin, morphine (2.5 mg/ml/kg), DMSO+exercise, Curcumin+exercise. * as comparison between saline and exercise, DMSO, Curcumin, morphine. Ψ<0.05 as comparison between morphine and DMSO+exercise, Curcumin+exercise.

**Figure 4:**
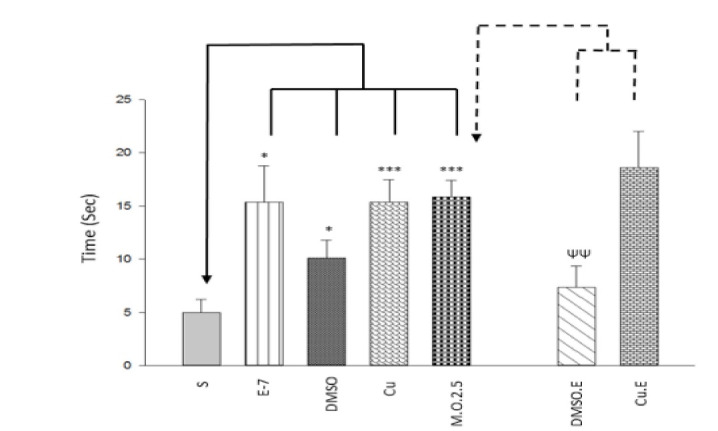
The effect of concurrent usage of exercise and Curcumin on spent time in the target quadrant. Subjects were divided to seven groups; saline, exercise (one week), DMSO, Curcumin, morphine (2.5 mg/ml/kg), DMSO+exercise, Curcumin+exercise. *P<0.05 and ***P<0.001 as comparison between saline and exercise, DMSO, Curcumin, morphine. ΨP<0.01 as comparison between morphine and DMSO+exercise, Curcumin+exercise.

**Figure 5:**
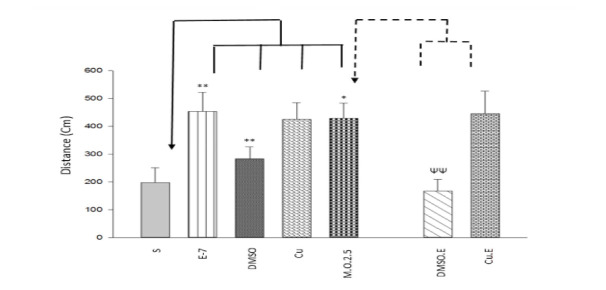
The effect of concurrent usage of exercise and Curcumin on moved distance in the target quadrant. Subjects were divided to seven groups: saline, exercise (one week), DMSO, Curcumin, morphine (2.5 mg/ml/kg), DMSO+exercise, Curcumin+exercise. *P<0.05 and ** P<0.01* as comparison between saline and exercise, DMSO, Curcumin, morphine. ΨΨP<0.01 as comparison between morphine and DMSO+exercise, Curcumin+exercise.

One way ANOVA and Tukey post hoc test showed that exercise, DMSO injection, and dependency to 2.5 mg/ml/kg morphine increased time (F [6,49]=4.686, P<0.05; Figure-3) and distance (F [6,49]=4.468, P<0.05; Figure-4) in target quadrant, however, Curcumin injection increased spent time (F [6,49]=4.686, P<0.05; Figure-3) but not moved distance in target quadrant (F [6,49]=4.468, P>0.05; Figure-4). Furthermore, in terms of dependency to 2.5 mg/ml/kg morphine, Independent T-test indicated that DMSO (but not Curcumin) injection with 1-week swimming decreased time (t=3.368, P<0.05; Figure-3) and distance (t=3.532, P<0.05; Figure-4) in the platform zone. As a result, DMSO injection with 1-week exercise can alter responses (spent time and moved distance) induced by morphine (2.5 mg/ml/kg) on spatial memory formation in the sensitization(Figure-5).

## Discussion

The findings revealed that DMSO + exercise and Curcumin + exercise increased escape latency and decreased swimming path length to find the hidden platform; that is, they decreased moving velocity and learning process induced by morphine (2.5 mg/ml/kg) in the sensitization period. The results indicated that exercise, DMSO, and Curcumin increased spent time and moved distance in the target quadrant; that is, they increased memory formation induced by 2.5 mg/ml/kg morphine. Moreover, DMSO + exercise decreased spent time and moved distance in platform quadrant that indicated it decreased memory formation induced by morphine, but Curcumin + exercise could return the effect of DMSO on cognition. Some researches show that DMSO does not affect memory [19, 24, 25]. It seems that the differences between those studies and the present study are because of the DMSO dosage (2.5 ml vs. 0.5 µl, 1 % or 4 µl/g). But some studies mentioned the positive effects of it on memory deficit. They indicated that DMSO was neuroprotective because of chemical and electrical changes induced by it, that consequently leads to synaptic alteration. Additionally, it can reduce Ca responding by N-Methyl-D-aspartic acid receptors to glutamate that gives protection against neuronal death induced by stimulus toxicity [26]. In agreement with our study, Shanmugasundaram *et al*. showed that DMSO (same as our dosage) impairs spatial memory and decreases synaptic plasticity [27]. Also, Budinich *et al*. demonstrated that DMSO (10- 15%) decreases swimming speed in MWM [28]. Moreover, the findings showed that DMSO + exercise decreased spatial memory, but Curcumin retrieved this effect, so a group of Curcumin + exercise had normal memory compare to morphine group. Previous researches showed that exercise increases endogen opioids such as beta-endorphins. So, it can be neuroprotective against morphine. Also, physical activity causes neurogenesis by BDNF, IGF-1, and increasing neurotransmitters such as DA, serotonin, etc. [29-36] that leads to better cognitive performance. However, some literature mentioned that swimming could act as a stress factor, and it can increase serum corticosterone that affects negatively on learning and memory process [1, 37, 38]. Stressor events activate cAMP response element-binding protein in nucleus accumbens that can affect cognition [39]. Physical stressors like intense exercise activate brain stem structures that lead to elevated activity of hypothalamic–pituitary–adrenal axis, chronic, and increasing corticotropin-releasing hormone secretion from the paraventricular nucleus in the hypothalamus [40, 41]. Chronic stress prevents DA from releasing in mesolimbic and catecholamines in the prelimbic brain [42]. It seems that short term (1 week) swim training was a stressor in our study that its combination with DMSO leads to spatial memory impairment. Maybe it needed to swim for longer period (more weeks) to appear positive effects of exercise. Curcumin decreases lipid oxidation, inflammation cytokines, free oxygen radicals, etc. [43-46] and increases BDNF, mitochondrial biogenesis, and neurogenesis [45] that leads to improvement in cognitive performance. Also, it affects the noradrenergic system and decreases mono amino oxidase that is a neuro disorder factor [37]. The results indicated that Curcumin could undo negative effects of the DMSO+ exercise combination on cognition. Curcumin could reduce the harmful effects of long term stress in memory and retention.

## Conclusion

As a solvent, DMSO had independent effects on memory that in combination with exercise lead to memory impairment. Suitable solvents have no significant effects on behavioral experiments. It seems that DMSO (with a dosage of 2.5 ml) isn’t an appropriate solvent for curcumin because of its unpredictable effects that may be undesirable. Hence, it should be used carefully.

## Acknowledgment

The authors would like to thank Dr. Mohammad Reza Zarrin Dast for his valuable comments, and personnel of the IranianNational Centerfor Addiction Study. This results as part of the Ph.D. dissertation of Laleh Elhampour in Islamic Azad University, Central Tehran Branch (No. 5017897).

## Conflict of Interest

We declare that there is no conflict of interest.
